# Clinicopathological features of male patients with breast cancer based on a nationwide registry database in Japan

**DOI:** 10.1007/s12282-022-01378-6

**Published:** 2022-06-22

**Authors:** Akihiko Shimomura, Masayuki Nagahashi, Hiraku Kumamaru, Kenjiro Aogi, Sota Asaga, Naoki Hayashi, Kotaro Iijima, Takayuki Kadoya, Yasuyuki Kojima, Makoto Kubo, Minoru Miyashita, Hiroaki Miyata, Naoki Niikura, Etsuyo Ogo, Kenji Tamura, Kenta Tanakura, Masayuki Yoshida, Yutaka Yamamoto, Shigeru Imoto, Hiromitsu Jinno

**Affiliations:** 1 Department of Breast and Medical Oncology, National Center for Global Health and Medicine, 1-21-1 Toyama, Shinjuku-ku, Tokyo, 162-8655 Japan; 2 Department of Breast and Endocrine Surgery, Hyogo Medical University, 1-1 Mukogawa-cho, Nishinomiya-shi, Hyogo 663-8501 Japan; 3 Department of Healthcare Quality Assessment, University of Tokyo, 7-3-1 Hongo, Bunkyo-ku, Tokyo, 113-8655 Japan; 4 National Clinical Database, Tokyo, Japan; 5 Department of Breast Oncology, National Hospital Organization Shikoku Cancer Center, Kou 160, Minamiumemotomachi, Matsuyama-shi, Ehime 791-0280 Japan; 6 Department of Breast Surgery, Kyorin University School of Medicine, 6-20-2 Shinkawa, Mitaka-shi, Tokyo 181-8611 Japan; 7 Department of Breast Surgical Oncology, St. Luke’s International Hospital, 9-1 Akashicho, Chuo-ku, Tokyo, 104-8560 Japan; 8 Department of Breast Oncology, Juntendo University, 3-1-3 Hongo, Bunkyo-ku, Tokyo, 113-8431 Japan; 9 Department of Surgical Oncology, Research Institute for Radiation Biology and Medicine, Hiroshima University, 1-2-3 Kasumi, Minami-ku, Hiroshima 734-0037 Japan; 10 Division of Breast and Endocrine Surgery, Department of Surgery, St. Marianna University School of Medicine, 2-16-1 Sugao, Miyamae-ku, Kawasaki-shi, Kanagawa 216-8511 Japan; 11 Department of Surgery and Oncology, Graduate School of Medical Sciences, Kyushu University, 3-1-1 Maidashi Higashi-ku, Fukuoka-shi, Fukuoka 812-8582 Japan; 12 Department of Breast and Endocrine Surgical Oncology, Tohoku University School of Medicine, Seiryo-machi, Aoba-ku, Sendai-shi, Miyagi 980-8574 Japan; 13 Department of Health Policy and Management, Keio University School of Medicine, 35 Shinanomachi, Shinjuku-ku, Tokyo, 160-8582 Japan; 14 Department of Breast Oncology, Tokai University School of Medicine, 143 Shimokasuya, Isehara-shi, Kanagawa 259-1193 Japan; 15 Department of Radiology, Kurume University School of Medicine, 67 Asahi-Machi, Kurume-shi, Fukuoka 830-0011 Japan; 16 Department of Medical Oncology, Shimane University Hospital, Shimane, Japan; 17 Division of Plastic and Reconstructive Surgery, Mitsui Memorial Hospital, 1 Kanda-Izumi-cho, Chiyoda-ku, Tokyo, 101-8643 Japan; 18 Department of Diagnostic Pathology, National Cancer Center Hospital, 5-1-1 Tsukiji, Chuo-ku, Tokyo, 104-0045 Japan; 19 Department of Breast and Endocrine Surgery, Kumamoto University Hospital, 1-1-1 Honjo, Chuo-ku, Kumamoto-shi, Kumamoto, 860-8556 Japan; 20 Department of Surgery, Teikyo University School of Medicine, 2-11-1 Kaga, Itabashi-ku, Tokyo, 173-8606 Japan

**Keywords:** Male breast cancer, National clinical database, Japanese

## Abstract

**Background:**

Male breast cancer (MBC) is rare; however, its incidence is increasing. There have been no large-scale reports on the clinicopathological characteristics of MBC in Japan.

**Methods:**

We investigated patients diagnosed with breast cancer in the Japanese National Clinical Database (NCD) between January 2012 and December 2018.

**Results:**

A total of 594,316 cases of breast cancer, including 3780 MBC (0.6%) and 590,536 female breast cancer (FBC) (99.4%), were evaluated. The median age at MBC and FBC diagnosis was 71 (45–86, 5–95%) and 60 years (39–83) (*p* < 0.001), respectively. MBC cases had a higher clinical stage than FBC cases: 7.4 vs. 13.3% stage 0, 37.2 vs. 44.3% stage I, 25.6 vs. 23.9% stage IIA, 8.8 vs. 8.4% stage IIB, 1.9 vs. 2.4% stage IIIA, 10.1 vs. 3.3% stage IIIB, and 1.1 vs. 1.3% stage IIIC (*p* < 0.001). Breast-conserving surgery was more frequent in FBC (14.6 vs. 46.7%, *p* = 0.02). Axillary lymph node dissection was more frequent in MBC cases (32.9 vs. 25.2%, *p* < 0.001). Estrogen receptor(ER)-positive disease was observed in 95.6% of MBC and 85.3% of FBC cases (*p* < 0.001). The HER2-positive disease rates were 9.5% and 15.7%, respectively (*p* < 0.001). Comorbidities were more frequent in MBC (57.3 vs. 32.8%) (*p* < 0.001). Chemotherapy was less common in MBC, while endocrine therapy use was similar in ER-positive MBC and FBC. Perioperative radiation therapy was performed in 14.3% and 44.3% of cases.

**Conclusion:**

Japanese MBC had an older age of onset, were more likely to be hormone receptor-positive disease, and received less perioperative chemotherapy than FBC.

**Supplementary Information:**

The online version contains supplementary material available at 10.1007/s12282-022-01378-6.

## Introduction

Breast cancer is rare among men, while it is the most common cancer among women, with approximately 91,605 cases (excluding intraepithelial cancer) in 2017 [1]. In 2017, the Japanese Breast Cancer Society’s Breast Cancer Registry reported 591 cases of male breast cancer (MBC), which continues to increase every year [2]. According to the Demographic Survey of the Ministry of Health, Labor and Welfare, the morbidity and mortality rates of both, MBC and female breast cancer (FBC) tend to increase; however, the trend is more moderate in MBC than FBC [3]. On the other hand, the lack of the data and information of MBC is the issue.

In Japan, cancer statistics are evaluated based on regional cancer registries; however, MBC is not documented, and its clinicopathological features have not been examined. In addition, the evaluation of biomarkers such as estrogen receptor (ER) and human epidermal growth factor receptor 2 (HER2) expression in the treatment of breast cancer is crucial yet lacking for MBC cases in Japan. According to previous reports from Western trials, MBC is characterized by older age and more hormone receptor-positive cases than FBC [4].

In 2011, the National Clinical Database (NCD), a nationwide system that links data collection to the first level of surgical specialization in the Japanese Surgical Board Certification System, adopted an annual web-based data collection system. In 2014, data on 1.6 million surgical procedures from more than 4,000 hospitals were collected [5]. Approximately 1.2 million cases are registered annually, representing more than 95% of the surgeries performed in Japan [6]. Many reports using the NCD have been published due to its reliable and abundant data [7–11]. Nevertheless, there are no large-scale reports on the clinicopathological characteristics of MBC in Japan. Understanding such characteristics is expected to be helpful for the treatment of this rare cancer. Thus, this study aimed to clarify the clinicopathological characteristics and identify the unmet needs of MBC cases in Japan.

## Methods

### Patient selection and data collection

We investigated patients diagnosed with breast cancer registered in the Japanese NCD between January 2012 and December 2018. This study was approved by the Institutional Review Board of the National Center for Global Health and Medicine (NCGM-G-003309-00) on September 6, 2019.

Robust patient variables, including age, sex, body mass index, performance status (PS), surgical procedure, family history, comorbidities, pathological factors, and perioperative systemic therapy, were obtained from the Japanese NCD.

ER and progesterone receptor (PgR) positivity were defined according to the ASCO/CAP 2010 guidelines [12], while HER2 positivity was defined according to the ASCO/CAP 2018 guidelines [13]. Subtypes were defined as follows: luminal type with HER2 negative, ER positive and/or PgR positive; luminal-HER2 type with HER2 positive, ER positive and/or PgR positive; HER2 type with HER2 positive, ER negative and PgR negative; triple negative breast cancer (TNBC) with HER2 negative, ER negative, and PgR negative.

Patients’ backgrounds were analyzed in total populations. TNM classification, histology, family history, and systemic treatment were analyzed according to each subtype. Comorbidity was collected from 2016. Thus it is analyzed data between 2016 and 2018.

### Statistical analysis

All statistical analyses were performed with SAS ver.9.4 (SAS Institute, Cary, NC, USA). Statistical comparisons of categorical variables were performed using chi-squared or Fisher’s exact probability tests. Continuous valuables were compared using the Wilcoxon rank-sum test. Continuous valuables are expressed using the median and interquartile range or average. Two-tailed *p* values < 0.05 were considered statistically significant.

## Results

### Patients

A total of 594,316 breast cancer cases, including 3780 cases of MBC (0.6%) and 590,536 cases of FBC (99.4%) were diagnosed between January 2012 and December 2018 in Japan (Table 1). The median ages at MBC or FBC diagnosis were 71 (45–86) and 60 years (39–83) (*p* < 0.001), respectively. Furthermore, 2.0% of the MBC cases and 5.6% of the FBC cases were under 40 years of age (*p* < 0.001). Bilateral disease was observed in 2.7% of the MBC cases and 9.7% of the FBC cases. Meanwhile, 97.0% of MBC cases and 90.1% of FBC cases were unilateral disease (*p* < 0.001). The median body mass index was higher in the MBC group than in the FBC group (23.1 vs. 22.3, *p* < 0.001). In addition, MBC cases tended to have higher clinical stages than FBC cases with resectable and/or locally advanced disease: 7.4 vs. 13.3% stage 0, 37.2 vs. 44.3% stage I, 25.6 vs. 23.9% stage IIA, 8.8 vs. 8.4% stage IIB, 1.9 vs. 2.4% stage IIIA, 10.1 vs. 3.3% stage IIIB, and 1.1 vs. 1.3% stage IIIC (*p* < 0.001). The frequency of de novo stage IV disease was similar between the two groups (2.1 vs. 2.0%). Over 95% of the cases in both groups underwent surgery (95.2 and 96.1%). Breast-conserving surgery was more frequent in FBC cases (14.6 vs. 46.7%, *p* = 0.02). Finally, axillary lymph node dissection with or without sentinel lymph node biopsy was more frequent in MBC cases (32.9 vs. 25.2%, *p* < 0.001).

**Table 1:** Patients characteristics

		Male		Female		*p* value
*N*		3780		590,536		
Age	Median, 5–95%	71 (45–86)		60 (39–83)		< 0.001
	< 20	17	0.4%	996	0.2%	< 0.001
	< 40	62	1.6%	31,683	5.4%	
	< 60	657	17.4%	252,212	42.7%	
	< 80	2263	59.9%	253,507	42.9%	
	80 and above	781	20.7%	52,138	8.8%	
Bilateral disease	Unilateral	3668	97.0%	531,864	90.1%	< 0.001
	Metachronous bilateral	36	1.0%	21,624	3.7%	
	Synchronous bilateral	63	1.7%	35,649	6.0%	
	Unknown	13	0.34%	1399	0.24%	
BMI kg/m^2^	Median, 5–95%	23.1 (17.9–29.6)		22.3 (17.6–30.5)		< 0.001
Clinical stage	Stage 0	280	7.4%	78,268	13.3%	< 0.001
	Stage I	1407	37.2%	261,509	44.3%	
	Stage IIA	969	25.6%	141,311	23.9%	
	Stage IIB	334	8.8%	49,409	8.4%	
	Stage IIIA	70	1.9%	14,346	2.4%	
	Stage IIIB	381	10.1%	19,432	3.3%	
	Stage IIIC	40	1.1%	7716	1.3%	
	Stage IV	81	2.1%	12,012	2.0%	
	Unknown	218	5.8%	16,533	2.8%	
Surgery	Yes	3599	95.2%	567,434	96.1%	0.02
	No	53	1.4%	6613	1.1%	
	Biopsy only	127	3.4%	16,451	2.8%	
	Unknown	1	0.0%	38	0.0%	
Breast surgery	Mastectomy	2835	75.0%	273,062	46.2%	< 0.001
	Breast-conserving surgery	552	14.6%	275,786	46.7%	
	Other/unknown	157	4.2%	10,121	1.7%	
	No breast surgery	55	1.5%	8465	1.4%	
Axillary surgery	SNB	1664	44.0%	345,504	58.5%	< 0.001
	SNB and axillary dissection	257	6.8%	35,544	6.0%	
	Axillary dissection	985	26.1%	113,255	19.2%	
	No axillary surgery	437	11.6%	44,263	7.5%	
	Sampling	39	1.0%	9096	1.5%	
	Other/unknown	4	0.1%	1186	0.2%	
	Missing	1	0.03%	90	0.02%	

*BMI* body mass index, *SNB* sentinel lymph node biopsy

### Pathological feature

**Fig. 1:**
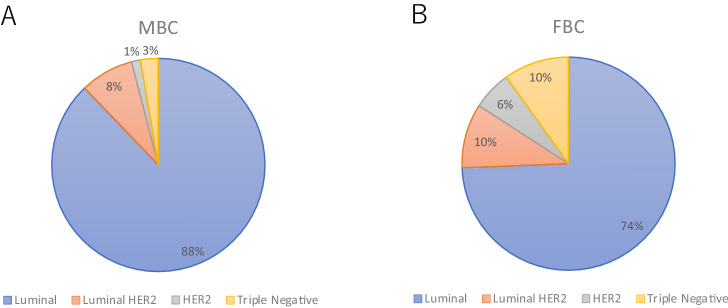
Distribution of each subtype in MBC (**a**) and FBC (**b**)

ER/PgR and HER2 statuses were available for 3003 (79.4%) MBC cases and 464,346 (78.6%) FBC cases. ER-positive disease was observed in 95.6% of MBC cases and 85.3% of FBC cases (*p* < 0.001). The PgR group had similar statistics between groups (90.4 vs. 72.9%, *p* < 0.001). The incidence of HER2-positive disease was 9.5% and 15.7% in MBC and FBC, respectively (*p* < 0.001). MBC exhibited larger tumors and more lymph node positivity (*p* < 0.001) but the same rate of M1 disease (1.4 vs. 1.3%, *p* = 0.37). Furthermore, invasive ductal carcinoma was more frequent in MBC cases (83.7 vs. 77.8%), and invasive lobular carcinoma was more frequent in FBC cases (1.3 vs. 4.6%) (*p* < 0.001). The distribution of the nuclear grade was similar between MBC and FBC cases (Table 2). Finally, the luminal subtype was more frequent in MBC cases (88 vs. 74%) (Fig. 1).

**Table 2:** Pathological features

		Male		Female		*p* value
N		3003		464,346		
T	T0	10	0.3%	1625	0.3%	< 0.001
	Tis	150	5.0%	45,176	9.7%	
	T1	1396	46.5%	235,664	50.8%	
	T2	987	32.9%	144,341	31.1%	
	T3	63	2.1%	13,883	3.0%	
	T4	362	12.1%	19,792	4.3%	
	Missing/unknown	35	1.2%	3863	0.8%	
N	Negative	2265	75.4%	381,398	82.1%	< 0.001
	Positive	685	22.8%	78,561	16.9%	
	Missing	53	1.8%	4387	0.9%	
M	M0	2917	97.1%	453,465	97.7%	0.37
	M1	43	1.4%	5823	1.3%	
	Missing	43	1.4%	5055	1.1%	
Histology	Invasive ductal	2514	83.7%	361,052	77.8%	< 0.001
	Invasive lobular	39	1.3%	21,421	4.6%	
	Others	447	14.9%	81,443	17.5%	
	Missing	3	0.1%	430	0.1%	
ER	Positive	2872	95.6%	387,500	83.5%	< 0.001
	Negarive	131	4.4%	76,802	16.5%	
	missing/not assessed	0	0.0%	44	0.0%	
PgR	Positive	2716	90.4%	338,617	72.9%	< 0.001
	Negarive	279	9.3%	125,050	26.9%	
	missing/not assessed	8	0.3%	679	0.1%	
HER2	Positive	284	9.5%	72,908	15.7%	< 0.001
	Negarive	2290	76.3%	333,601	71.8%	
	missing/not assessed	429	14.3%	57,837	12.5%	

*ER* estrogen receptor, *PgR* progesterone receptor, *HER2* human epidermal growth factor-2

### Family history of cancer and comorbidity

Patients with a family history of cancer were less likely to have MBC than FBC, regardless of the subtype (*p* < 0.001) (Tables 3). Missing/unknown family history was more frequent in patients with MBC compared with patients with FBC.

**Table 3:** Family history

	Male		Female		*p* value
N	3003		464,346		
Present	329	11.0%	63,058	13.6%	< 0.001
Absent	2339	77.9%	366,917	79.0%	
Missing /unknown	335	11.2%	34,371	7.4%	

Comorbidities are recorded in Table 4. The proportion of patients with comorbidities was 57.3% for MBC and 32.8% for FBC (*p* < 0.001). The most frequent comorbidities of MBC were hypertension (35.1%), diabetes (14.7%), other malignancies (11.7%), neuro/peripheral vascular disease (7.9%), and coronary artery disease (6.9%).

**Table 4:** Comorbidities

		Male		Female		*p* value
N		1781		276,341		
Comorbidities	Yes	1021	57.3%	90,602	32.8%	< 0.001
	No	760	42.7%	185,734	67.2%	
	Missing	0	0.0%	5	0.002%	
Coronary artery disease	Present	123	6.9%	4999	1.8%	< 0.001
Neuro/peripheral vascular disease	Present	140	7.9%	8265	3.0%	< 0.001
Other malignancy	Present	209	11.7%	13,784	5.0%	< 0.001
Congestive heart failure	Present	40	2.2%	2171	0.8%	< 0.001
COPD	Present	40	2.2%	1067	0.4%	< 0.001
Collagen disease	Present	10	0.6%	2564	0.9%	0.1
Liver disease	Present	67	3.8%	4053	1.5%	< 0.001
Diabetes	Present	262	14.7%	19,893	7.2%	< 0.001
Hypertension	Present	626	35.1%	60,932	22.0%	< 0.001
Renal disease	Present	84	4.7%	3853	1.4%	< 0.001

*COPD* chronic obstructive pulmonary disease

### Systemic treatment

Neoadjuvant systemic therapy and adjuvant systemic therapy were less frequent in MBC cases than FBC cases (8.9 vs. 11.9%, *p* < 0.001; 82.7 vs. 86.3%, *p* < 0.001) (Table 5). Chemotherapy was less commonly administered in MBC cases (22.0 vs. 32.7%, *p* < 0.001), while endocrine therapy was more frequent in MBC cases (76.0 vs. 67.9%, *p* < 0.001). Specifically, the administration of chemotherapy in MBC vs. FBC cases according to subtype was 19.2 vs. 23.7% for Luminal, 47.1 vs. 60.4% for Luminal-HER2, 40.0 vs. 62.6% for HER2, 57.3 vs. 69.9% for TNBC. Meanwhile, endocrine therapy administration was similar in Luminal and Luminal-HER2 MBC and FBC cases (84.9 vs. 83.9%, 68.0 vs. 72.5%, respectively) (Table S4). Anthracycline, taxane, and anti-HER2 drug therapy was less frequent in MBC cases compared to FBC cases (13.0 vs. 20.9%, 10.1 vs. 18.0%, and 4.9 vs. 10.2%, respectively, *p* < 0.001).

**Table 5:** Systemic treatment

			Male		Female		*p* value
*N*			3003		464,346		
Neoadjuvant systemic therapy	Received	Yes	266	8.9%	55,197	11.9%	< 0.001
	Not received	No	2730	90.9%	408,137	87.9%	
	Unknown	Missing	7	0.2%	1012	0.2%	
Adjuvant systemic therapy	Received	Yes	2484	82.7%	400,831	86.3%	< 0.001
	Not received	No	457	15.2%	53,250	11.5%	
	Unknown	Missing	62	2.1%	10,265	2.2%	
Treatment detail	Endocrine therapy	Yes	2282	76.0%	315,141	67.9%	< 0.001
	Chemotherapy	Yes	662	22.0%	151,839	32.7%	< 0.001
Chemotherapy regimen	Anthracycline	Yes	389	13.0%	96,962	20.9%	< 0.001
	Taxane	Yes	304	10.1%	83,444	18.0%	< 0.001
	anti-HER2	Yes	148	4.9%	47,249	10.2%	< 0.001

### Radiation therapy

Perioperative radiation therapy was performed in 14.3% of MBC cases and 44.3% of FBC cases (Table 6).

**Table 6:** Radiation therapy

	Male		Female		*p* value
N	3003		464,346		
Yes	430	14.3%	205,674	44.3%	< 0.001
No	2499	83.2%	245,156	52.8%	
Missing	74	2.5%	13,516	5.5%	

## Discussion

To the best of our knowledge, this is the first study to report the real-world clinicopathological characteristics and treatment trends of Japanese male patients with breast cancer based on a nationwide registry database. Approximately, 600,000 patients with breast cancer were included in the study, and the frequency of male breast cancer was comparable to previous reports from Western countries at 0.6% [14, 15]. Furthermore, similar to previous reports from Western countries, the median age of MBC cases was more than 10 years higher than that of FBC cases [16–20]. The frequency of relatively advanced stage II or III breast cancer was higher in MBC than FBC, and this tendency was similar with pathological stages (Tables S1, S2a). This may be because males have much smaller breast tissue than females; thus, breast cancer in males easily invade the skin and pectoral muscles [19, 21]. In addition, because males rarely visit breast oncologists due to a lack of understanding of breast cancer, they may believe that they do not have a risk of breast cancer. Nevertheless, the frequency of de novo stage IV disease was similar in MBC and FBC.

The breast-conserving surgery rate for MBC was 14.6%, which was lower than that of FBC but higher than that reported in Western countries. This may be due to tumors within 2 cm in size being relatively higher in Japanese MBC [4, 22]. Moreover, axillary lymph node dissection was more common in MBC, which may be due to the higher incidence of node-positive breast cancer in males. Additionally, similar to previous findings, MBC was more likely to be hormone receptor positive and less likely to be HER2 positive than FBC (Table S2b). The luminal subtype was the most common at 87.8%, which is consistent with previous reports [4, 23]. The frequency of the HER2-type and TNBC was slightly higher in our study than that of previous reports, suggesting that the biology may be different in Europe and the United States than in Japan [4, 23–25]. A positive family history of breast cancer was more common in FBC, while family history tended to be unknown in MBC cases (Table S3). Thus, clinicians may be taking insufficient family history of breast cancer for males.

Comorbidities were more common in the MBC group. Cardiovascular disease was the most frequent comorbidity, and the frequency of other malignancies was high (11.7%) in the patients with MBC (Table 4). In addition to the higher median age of MBC cases, hereditary tumor syndromes, such as hereditary breast and ovarian cancers, may be a cause of the higher rate of comorbidities and malignancies [25–27]. However, there were no data on detailed other malignancies in NCD.

Neoadjuvant systemic treatment was administered in less than 10% of MBC and FBC cases, and no significant difference of frequency was observed (Table S4). The frequency of endocrine therapy as a systemic treatment was similar between MBC and FBC cases; however, the frequency of chemotherapy with anthracyclines, taxanes, and anti-HER2 agents was lower in MBC. This suggests that patients’ general condition, performance status, and/or cardiovascular complications may influence oncologists’ decision-making with patients. The number of MBC cases who received radiotherapy was approximately one-third that of FBC cases, which could be because most MBC cases undergo mastectomy. Nevertheless, the proportion of patients who underwent BCS received radiation was slight in MBC. Furthermore, some patients eligible for post mastectomy radiation therapy, such as pN2 and pN3, also did not receive radiation. This may be related to older age and more comorbidities in Japanese MBC.

Real-world data analyses, such as the current study, boast a high number of patients. Our study included approximately 600,000 patients, which is much higher than of other retrospective studies. This high number of patients aids in the understanding of trends of clinicopathological features and treatment of MBC. Nevertheless, this study had several limitations. First, there was a risk of bias due to the retrospective nature of the study. Second, most databases, including the NCD, have missing data; thus, the true percentage of each value may not be reflected. Further analysis with a large cohort is required to obtain more reliable evidence. Addition to it, the NCD data are registered primarily by breast surgeons. Therefore, de novo stage IV data may be less available than the reality. Third, the NCD does not provide enough data on long-term survival, inhibiting the comparison of survival outcomes of MBC and FBC. Further studies should include additional histopathological and clinical data from the same cohort to obtain stronger conclusions, and such a study would be helpful in conducting clinical trials on MBC.

In conclusion, Japanese MBC had an older age of onset, were more likely to be hormone receptor-positive disease, and received less perioperative chemotherapy than FBC. This is the first comprehensive analysis using real-word data from a nationwide registry database in Japan of clinicopathological features and treatment trends in Japanese MBC cases. Further prospective studies are needed to evaluate the most suitable treatment strategy for MBC in Japan.

## Supplementary Information

Below is the link to the electronic supplementary material.

Supplementary file1 (DOCX 76 KB)
