# Supplementary material for: Clinicopathological features of male patients with breast cancer based on a nationwide registry database in Japan
Source: Breast Cancer. 2022 Jun 22;29(6):985–92. doi: 10.1007/s12282-022-01378-6 (PMC9587939; doi:10.1007/s12282-022-01378-6)
Supplement: Supplementary file 1 — Supplementary file1 (DOCX 76 KB) [file 12282_2022_1378_MOESM1_ESM.docx]

Table S1. Pathological stage

|  | Male | | Female | |
| --- | --- | --- | --- | --- |
| N | 3780 | | 590536 | |
| Stage 0 | 98 | 2.6% | 38850 | 6.6% |
| Stage I | 1140 | 30.2% | 228799 | 38.7% |
| Stage IIA | 771 | 20.4% | 120269 | 20.4% |
| Stage IIB | 302 | 8.0% | 44367 | 7.5% |
| Stage IIIA | 147 | 3.9% | 22998 | 3.9% |
| Stage IIIB | 210 | 5.6% | 17069 | 2.9% |
| Stage IIIC | 102 | 2.7% | 10533 | 1.8% |
| Stage IV | 81 | 2.1% | 12012 | 2.0% |
| Stage undefined | 829 | 21.9% | 95639 | 16.2% |

Table S2A. Details of pathological features

|  |  | Male | | Female | |
| --- | --- | --- | --- | --- | --- |
| N |  | 3003 | | 464346 | |
| T | T0 | 10 | 0.3% | 1625 | 0.3% |
|  | Tis | 150 | 5.0% | 45176 | 9.7% |
|  | T1 | 1396 | 46.5% | 235664 | 50.8% |
|  | T2 | 987 | 32.9% | 144341 | 31.1% |
|  | T3 | 63 | 2.1% | 13883 | 3.0% |
|  | T4 | 362 | 12.1% | 19792 | 4.3% |
|  | Missing/unknown | 35 | 1.2% | 3863 | 0.8% |
| T size | <0.5cm | 169 | 5.6% | 46929 | 10.1% |
|  | <1.0cm | 250 | 8.3% | 58826 | 12.7% |
|  | <2.0cm | 1174 | 39.1% | 165455 | 35.6% |
|  | <3.0cm | 790 | 26.3% | 93714 | 20.2% |
|  | <5.0cm | 422 | 14.1% | 57182 | 12.3% |
|  | over 5.0cm | 123 | 4.1% | 27602 | 5.9% |
|  | Missing | 75 | 2.5% | 14638 | 3.2% |
| N | N0 | 2265 | 75.4% | 381398 | 82.1% |
|  | N1 | 594 | 19.8% | 62979 | 13.6% |
|  | N2 | 58 | 1.9% | 9035 | 1.9% |
|  | N3 | 33 | 1.1% | 6537 | 1.4% |
|  | Missing | 53 | 1.8% | 4387 | 0.9% |
| M | M0 | 2917 | 97.1% | 453465 | 97.7% |
|  | M1 | 43 | 1.4% | 5823 | 1.3% |
|  | Missing | 43 | 1.4% | 5055 | 1.1% |
| Histology | Invasive ductal | 2514 | 83.7% | 361052 | 77.8% |
|  | Invasive lobular | 39 | 1.3% | 21421 | 4.6% |
|  | Others | 447 | 14.9% | 81443 | 17.5% |
|  | Missing | 3 | 0.1% | 430 | 0.1% |
| ER | 1-9% | 70 | 2.3% | 16572 | 3.6% |
|  | 10% and above | 2802 | 93.3% | 370928 | 79.9% |
|  | <1% | 131 | 4.4% | 82216 | 17.7% |
|  | missing / not assessed | 0 | 0.0% | 44 | 0.0% |
| PgR | 1-9% | 197 | 6.6% | 38798 | 8.4% |
|  | 10% and above | 2519 | 83.9% | 299819 | 64.6% |
|  | <1% | 279 | 9.3% | 125050 | 26.9% |
|  | missing / not assessed | 8 | 0.3% | 679 | 0.1% |
| HER2 IHC | 0 | 1203 | 40.1% | 171187 | 36.9% |
|  | 1+ | 1107 | 36.9% | 164272 | 35.4% |
|  | 2+ | 463 | 15.4% | 59989 | 12.9% |
|  | 3+ | 154 | 5.1% | 56605 | 12.2% |
|  | Missing / not assessed | 75 | 2.5% | 12268 | 2.6% |
| HER2 FISH | positive | 135 | 4.5% | 20612 | 4.4% |
|  | negative | 634 | 21.1% | 83579 | 18.0% |
|  | missing / non assessed | 2234 | 74.4% | 360155 | 77.6% |
| Number of data years 2013-2018 | | 2641 | 87.9% | 406624 | 87.6% |
| Nuclear grade | 1 | 933 | 31.1% | 158020 | 34.0% |
|  | 2 | 953 | 31.7% | 124438 | 26.8% |
|  | 3 | 498 | 16.6% | 82476 | 17.8% |
|  | Missing / unknown | 257 | 8.6% | 41690 | 9.0% |

Abbreviations: ER; estrogen receptor, PgR; progesterone receptor, HER2: human epidermal growth factor receptor-2, IHC; immunohistochemistry, FISH; fluorescence in-situ hybridization

Table S2B. Pathological feature by subtypes

|  |  | Luminal | | | | | Luminal HER2 | | | | | HER2 | | | | | Triple Negative | | | | |
| --- | --- | --- | --- | --- | --- | --- | --- | --- | --- | --- | --- | --- | --- | --- | --- | --- | --- | --- | --- | --- | --- |
|  |  | 347931 | | | | | 45710 | | | | | 27482 | | | | | 46226 | | | | |
|  |  | Male | | Female | | P value | Male | | Female | | P value | Male | | Female | | P value | Male | | Female | | P value |
| Num |  | 2637 | 1% | 345294 | 99.2% |  | 244 | 0.5% | 45466 | 99.5% |  | 40 | 0.1% | 27442 | 99.9% |  | 82 | 0.2% | 46144 | 99.8% |  |
| T | T0 | 8 | 0.3% | 1175 | 0.3% | <0.001 | 1 | 0.4% | 165 | 0.4% | <0.001 | 1 | 2.5% | 143 | 0.5% | 0.27 | 0 | 0.0% | 142 | 0.3% | 0.02 |
|  | Tis | 129 | 4.9% | 31931 | 9.2% |  | 10 | 4.1% | 5470 | 12.0% |  | 7 | 17.5% | 5081 | 18.5% |  | 4 | 4.9% | 2694 | 5.8% |  |
|  | T1 | 1259 | 47.7% | 190367 | 55.1% |  | 98 | 40.2% | 18956 | 41.7% |  | 14 | 35.0% | 8539 | 31.1% |  | 25 | 30.5% | 17802 | 38.6% |  |
|  | T2 | 858 | 32.5% | 98126 | 28.4% |  | 87 | 35.7% | 16628 | 36.6% |  | 11 | 27.5% | 10176 | 37.1% |  | 31 | 37.8% | 19411 | 42.1% |  |
|  | T3 | 47 | 1.8% | 8313 | 2.4% |  | 7 | 2.9% | 1668 | 3.7% |  | 2 | 5.0% | 1468 | 5.3% |  | 7 | 8.5% | 2434 | 5.3% |  |
|  | T4 | 307 | 11.6% | 12652 | 3.7% |  | 36 | 14.8% | 2158 | 4.7% |  | 5 | 12.5% | 1712 | 6.2% |  | 14 | 17.1% | 3270 | 7.1% |  |
|  | Missing/unknown | 29 | 1.1% | 2730 | 0.8% |  | 5 | 2.0% | 419 | 0.9% |  | 0 | 0.0% | 323 | 1.2% |  | 1 | 1.2% | 391 | 0.8% |  |
| T size | <0.5cm | 152 | 5.8% | 32336 | 9.4% | <0.001 | 8 | 3.3% | 5565 | 12.2% | <0.001 | 6 | 15.0% | 5203 | 19.0% | 0.94 | 3 | 3.7% | 3825 | 8.3% | 0.25 |
|  | <1.0cm | 223 | 8.5% | 47713 | 13.8% |  | 19 | 7.8% | 4425 | 9.7% |  | 3 | 7.5% | 2421 | 8.8% |  | 5 | 6.1% | 4267 | 9.2% |  |
|  | <2.0cm | 1043 | 39.6% | 130167 | 37.7% |  | 87 | 35.7% | 14558 | 32.0% |  | 12 | 30.0% | 6697 | 24.4% |  | 32 | 39.0% | 14033 | 30.4% |  |
|  | <3.0cm | 692 | 26.2% | 68180 | 19.7% |  | 75 | 30.7% | 9813 | 21.6% |  | 8 | 20.0% | 5153 | 18.8% |  | 15 | 18.3% | 10568 | 22.9% |  |
|  | <5.0cm | 365 | 13.8% | 39415 | 11.4% |  | 38 | 15.6% | 6244 | 13.7% |  | 5 | 12.5% | 4007 | 14.6% |  | 14 | 17.1% | 7516 | 16.3% |  |
|  | over 5.0cm | 102 | 3.9% | 18308 | 5.3% |  | 7 | 2.9% | 2897 | 6.4% |  | 4 | 10.0% | 2179 | 7.9% |  | 10 | 12.2% | 4218 | 9.1% |  |
|  | Missing | 60 | 2.3% | 9175 | 2.7% |  | 10 | 4.1% | 1964 | 4.3% |  | 2 | 5.0% | 1782 | 6.5% |  | 3 | 3.7% | 1717 | 3.7% |  |
| N | N0 | 2020 | 76.6% | 293194 | 84.9% | <0.001 | 160 | 65.6% | 34844 | 76.6% | <0.001 | 30 | 75.0% | 19910 | 72.6% | 0.38 | 55 | 67.1% | 33450 | 72.5% | 0.43 |
|  | N1 | 505 | 19.2% | 40461 | 11.7% |  | 67 | 27.5% | 7998 | 17.6% |  | 5 | 12.5% | 5421 | 19.8% |  | 17 | 20.7% | 9099 | 19.7% |  |
|  | N2 | 40 | 1.5% | 5169 | 1.5% |  | 10 | 4.1% | 1267 | 2.8% |  | 2 | 5.0% | 978 | 3.6% |  | 6 | 7.3% | 1621 | 3.5% |  |
|  | N3 | 24 | 0.9% | 3296 | 1.0% |  | 3 | 1.2% | 935 | 2.1% |  | 3 | 7.5% | 852 | 3.1% |  | 3 | 3.7% | 1454 | 3.2% |  |
|  | Missing | 48 | 1.8% | 3174 | 0.9% |  | 4 | 1.6% | 422 | 0.9% |  | 0 | 0.0% | 281 | 1.0% |  | 1 | 1.2% | 510 | 1.1% |  |
| M | M0 | 2567 | 97.3% | 338185 | 97.9% | 0.09 | 237 | 97.1% | 44163 | 97.1% | 0.49 | 39 | 97.5% | 26462 | 96.4% | 0.47 | 74 | 90.2% | 44655 | 96.8% | <0.001 |
|  | M1 | 33 | 1.3% | 3421 | 1.0% |  | 3 | 1.2% | 834 | 1.8% |  | 0 | 0.0% | 648 | 2.4% |  | 7 | 8.5% | 920 | 2.0% |  |
|  | Missing | 37 | 1.4% | 3688 | 1.1% |  | 4 | 1.6% | 469 | 1.0% |  | 1 | 2.5% | 329 | 1.2% |  | 1 | 1.2% | 569 | 1.2% |  |
| Histology | Invasive ductal | 2217 | 84.1% | 269927 | 78.2% | <0.001 | 214 | 87.7% | 36769 | 80.9% | 0.01 | 29 | 72.5% | 20188 | 73.6% | 0.65 | 54 | 65.9% | 34168 | 74.0% | 0.23 |
|  | Invasive lobular | 33 | 1.3% | 18519 | 5.4% |  | 1 | 0.4% | 1036 | 2.3% |  | 1 | 2.5% | 282 | 1.0% |  | 4 | 4.9% | 1584 | 3.4% |  |
|  | Others | 384 | 14.6% | 56568 | 16.4% |  | 29 | 11.9% | 7605 | 16.7% |  | 10 | 25.0% | 6928 | 25.2% |  | 24 | 29.3% | 10342 | 22.4% |  |
|  | Missing | 3 | 0.1% | 280 | 0.1% |  | 0 | 0.0% | 56 | 0.1% |  | 0 | 0.0% | 44 | 0.2% |  | 0 | 0.0% | 50 | 0.1% |  |
| ER | 1-9% | 59 | 2.2% | 11036 | 3.2% | 0.005 | 11 | 4.5% | 5536 | 12.2% | 0.004 | 0 | 0.0% | 0 | 0.0% | - | **0** | 0.0% | 0 | 0.0% | - |
|  | 10% and above | 2572 | 97.5% | 332101 | 96.2% |  | 230 | 94.3% | 38827 | 85.4% |  | 0 | 0.0% | 0 | 0.0% |  | 0 | 0.0% | 0 | 0.0% |  |
|  | <1% | 6 | 0.2% | 7535 | 2.2% |  | 3 | 1.2% | 1095 | 2.4% |  | 40 | 100.0% | 27442 | 100.0% |  | 82 | 100.0% | 46144 | 100.0% |  |
|  | missing / not assessed | 0 | 0.0% | 36 | 0.0% |  | 0 | 0.0% | 8 | 0.0% |  | 0 | 0.0% | 0 | 0.0% |  | 0 | 0.0% | 0 | 0.0% |  |
| PgR | 1-9% | 175 | 6.6% | 31564 | 9.1% | <0.001 | 22 | 9.0% | 7234 | 15.9% | <0.001 | 0 | 0.0% | 0 | 0.0% | - | **0** | 0.0% | 0 | 0.0% |  |
|  | 10% and above | 2340 | 88.7% | 274645 | 79.5% |  | 179 | 73.4% | 25174 | 55.4% |  | 0 | 0.0% | 0 | 0.0% |  | 0 | 0.0% | 0 | 0.0% |  |
|  | <1% | 115 | 4.4% | 38507 | 11.2% |  | 42 | 17.2% | 12957 | 28.5% |  | 40 | 100.0% | 27442 | 100.0% |  | 82 | 100.0% | 46144 | 100.0% |  |
|  | missing / not assessed | 7 | 0.3% | 578 | 0.2% |  | 1 | 0.4% | 101 | 0.2% |  | 0 | 0.0% | 0 | 0.0% |  | 0 | 0.0% | 0 | 0.0% |  |
| HER2 IHC | 0 | 1144 | 43.4% | 145197 | 42.1% | 0.04 | 10 | 4.1% | 491 | 1.1% | <0.001 | 0 | 0.0% | 86 | 0.3% | 0.67 | 49 | 59.8% | 25413 | 55.1% | 0.40 |
|  | 1+ | 1077 | 40.8% | 148612 | 43.0% |  | 10 | 4.1% | 1141 | 2.5% |  | 0 | 0.0% | 140 | 0.5% |  | 20 | 24.4% | 14379 | 31.2% |  |
|  | 2+ | 350 | 13.3% | 42270 | 12.2% |  | 97 | 39.8% | 10248 | 22.5% |  | 5 | 12.5% | 2205 | 8.0% |  | 11 | 13.4% | 5266 | 11.4% |  |
|  | 3+ | 0 | 0.0% | 0 | 0.0% |  | 122 | 50.0% | 32153 | 70.7% |  | 32 | 80.0% | 24452 | 89.1% |  | 0 | 0.0% | 0 | 0.0% |  |
|  | Missing / not assessed | 66 | 2.5% | 9215 | 2.7% |  | 5 | 2.0% | 1433 | 3.2% |  | 2 | 5.0% | 534 | 1.9% |  | 2 | 2.4% | 1086 | 2.4% |  |
| HER2 FISH | positive | 0 | 0.0% | 0 | 0.0% | - | 126 | 51.6% | 15803 | 34.8% | <0.001 | 9 | 22.5% | 4809 | 17.5% | 0.75 | 0 | 0.0% | 0 | 0.0% | - |
|  | negative | 610 | 23.1% | 73266 | 21.2% |  | 2 | 0.8% | 614 | 1.4% |  | 0 | 0.0% | 219 | 0.8% |  | 22 | 26.8% | 9480 | 20.5% |  |
|  | missing / non assessed | 2027 | 76.9% | 272028 | 78.8% |  | 116 | 47.5% | 29049 | 63.9% |  | 31 | 77.5% | 22414 | 81.7% |  | 60 | 73.2% | 36664 | 79.5% |  |
| Number of data years 2013-2018 | | 2324 |  |  | 303246 |  | 211 |  |  | 39698 |  | 37 |  |  | 23606 |  | 69 |  |  | 40074 |  |
| Nuclear grade | 1 | 869 | 33.0% | 141940 | 41.1% | <0.001 | 46 | 18.9% | 8570 | 18.8% | 0.09 | 7 | 17.5% | 2233 | 8.1% | 0.35 | 11 | 13.4% | 5277 | 11.4% | 0.02 |
|  | 2 | 848 | 32.2% | 95859 | 27.8% |  | 82 | 33.6% | 13007 | 28.6% |  | 7 | 17.5% | 5943 | 21.7% |  | 16 | 19.5% | 9629 | 20.9% |  |
|  | 3 | 385 | 14.6% | 37107 | 10.7% |  | 70 | 28.7% | 13407 | 29.5% |  | 17 | 42.5% | 11782 | 42.9% |  | 26 | 31.7% | 20180 | 43.7% |  |
|  | Missing / unknown | 222 | 8.4% | 28340 | 8.2% |  | 13 | 5.3% | 4714 | 10.4% |  | 6 | 15.0% | 3648 | 13.3% |  | 16 | 19.5% | 4988 | 10.8% |  |

Abbreviations: ER; estrogen receptor, PgR; progesterone receptor, HER2: human epidermal growth factor receptor-2, IHC; immunohistochemistry, FISH; fluorescence in-situ hybridization

Table S3. Family history by subtype

|  | Luminal | | | | | Luminal HER2 | | | | | HER2 | | | | | Triple Negative | | | | | |
| --- | --- | --- | --- | --- | --- | --- | --- | --- | --- | --- | --- | --- | --- | --- | --- | --- | --- | --- | --- | --- | --- |
|  | Male | | Female | | P value | Male | | Female | | P value | Male | | Female | | P value | | Male | | Female | | P value |
| N | 2637 | | 345294 | |  | 244 | | 45466 | |  | 40 | | 27442 | |  | | 82 | | 46144 | |  |
| Present | 298 | 11.3% | 47697 | 13.8% | <0.001 | 23 | 9.4% | 6103 | 13.4% | 0.07 | 3 | 7.5% | 3252 | 11.9% | 0.21 | | 5 | 6.1% | 6006 | 13.0% | 0.13 |
| Absent | 2045 | 77.6% | 272780 | 79.0% |  | 195 | 79.9% | 35764 | 78.7% |  | 31 | 77.5% | 21973 | 80.1% |  |  | 68 | 82.9% | 36400 | 78.9% |  |
| Missing / unknown | 294 | 11.1% | 24817 | 7.2% |  | 26 | 10.7% | 3599 | 7.9% |  | 6 | 15.0% | 2217 | 8.1% |  |  | 9 | 11.0% | 3738 | 8.1% |  |

Table S4 Systemic therapy by subtypes

|  |  |  | Luminal | | | | Luminal HER2 | | | | HER2 | | | | Triple Negative | | | |
| --- | --- | --- | --- | --- | --- | --- | --- | --- | --- | --- | --- | --- | --- | --- | --- | --- | --- | --- |
|  |  |  | Male | | Female | | Male | | Female | | Male | | Female | | Male | | Female | |
|  | N |  | 2637 | | 345294 | | 244 | | 45466 | | 40 | | 27442 | | 82 | | 46144 | |
| Noadjuvant therapy | Received |  | 217 | 8.2% | 33225 | 9.6% | 32 | 13.1% | 7469 | 16.4% | 6 | 15.0% | 5034 | 18.3% | 11 | 13.4% | 9469 | 20.5% |
|  | Not received |  | 2413 | 91.5% | 311334 | 90.2% | 212 | 86.9% | 37897 | 83.4% | 34 | 85.0% | 22342 | 81.4% | 71 | 86.6% | 36564 | 79.2% |
|  | Unknown |  | 7 | 0.3% | 735 | 0.2% | 0 | 0.0% | 100 | 0.2% | 0 | 0.0% | 66 | 0.2% | 0 | 0.0% | 111 | 0.2% |
|  | Endocrine therapy | Yes | 123 | 4.7% | 14178 | 4.1% | 12 | 4.9% | 1477 | 3.2% | 0 | 0.0% | 189 | 0.7% | 1 | 1.2% | 432 | 0.9% |
|  |  | No | 94 | 3.6% | 19038 | 5.5% | 20 | 8.2% | 5987 | 13.2% | 6 | 15.0% | 4844 | 17.7% | 10 | 12.2% | 9036 | 19.6% |
|  |  | Missing | 0 | 0.0% | 9 | 0.0% | 0 | 0.0% | 5 | 0.0% | 0 | 0.0% | 1 | 0.0% | 0 | 0.0% | 1 | 0.0% |
|  | Chemotherapy | Yes | 97 | 3.7% | 19998 | 5.8% | 22 | 9.0% | 6264 | 13.8% | 5 | 12.5% | 4748 | 17.3% | 10 | 12.2% | 9067 | 19.6% |
|  |  | No | 120 | 4.6% | 13225 | 3.8% | 10 | 4.1% | 1204 | 2.6% | 1 | 2.5% | 286 | 1.0% | 1 | 1.2% | 402 | 0.9% |
|  |  | Missing | 0 | 0.0% | 2 | 0.0% | 0 | 0.0% | 1 | 0.0% | 0 | 0.0% | 0 | 0.0% | 0 | 0.0% | 0 | 0.0% |
|  | Regimen | Anthracycline | 79 | 3.0% | 16613 | 4.8% | 17 | 7.0% | 4733 | 10.4% | 5 | 12.5% | 3483 | 12.7% | 7 | 8.5% | 7749 | 16.8% |
|  |  | Taxane | 71 | 2.7% | 15545 | 4.5% | 16 | 6.6% | 5264 | 11.6% | 4 | 10.0% | 4003 | 14.6% | 7 | 8.5% | 7199 | 15.6% |
|  |  | antiHER2 | 5 | 0.2% | 2071 | 0.6% | 13 | 5.3% | 4805 | 10.6% | 3 | 7.5% | 3970 | 14.5% | 1 | 1.2% | 599 | 1.3% |
|  |  | other | 17 | 0.6% | 2823 | 0.8% | 1 | 0.4% | 820 | 1.8% | 0 | 0.0% | 666 | 2.4% | 2 | 2.4% | 1522 | 3.3% |
| Adjuvant therapy | Received |  | 2215 | 84.0% | 308203 | 89.3% | 209 | 85.7% | 39779 | 87.5% | 19 | 47.5% | 20277 | 73.9% | 41 | 50.0% | 32572 | 70.6% |
|  | Not received |  | 370 | 14.0% | 29630 | 8.6% | 30 | 12.3% | 4616 | 10.2% | 16 | 40.0% | 6531 | 23.8% | 41 | 50.0% | 12473 | 27.0% |
|  | Unknown/No surgery |  | 52 | 2.0% | 7461 | 2.2% | 5 | 2.0% | 1071 | 2.4% | 5 | 12.5% | 634 | 2.3% | 0 | 0.0% | 1099 | 2.4% |
|  | Endocrine therapy | Yes | 2086 | 79.1% | 278940 | 80.8% | 165 | 67.6% | 29421 | 64.7% | 3 | 7.5% | 1078 | 3.9% | 2 | 2.4% | 2018 | 4.4% |
|  |  | No | 129 | 4.9% | 29228 | 8.5% | 44 | 18.0% | 10348 | 22.8% | 16 | 40.0% | 19196 | 70.0% | 39 | 47.6% | 30540 | 66.2% |
|  |  | Missing | 0 | 0.0% | 35 | 0.0% | 0 | 0.0% | 10 | 0.0% | 0 | 0.0% | 3 | 0.0% | 0 | 0.0% | 14 | 0.0% |
|  | Chemotherapy | Yes | 409 | 15.5% | 61965 | 17.9% | 93 | 38.1% | 21199 | 46.6% | 11 | 27.5% | 12431 | 45.3% | 37 | 45.1% | 23201 | 50.3% |
|  |  | No | 1806 | 68.5% | 246193 | 71.3% | 116 | 47.5% | 18672 | 41.1% | 8 | 20.0% | 7844 | 28.6% | 4 | 4.9% | 9361 | 20.3% |
|  |  | Missing | 0 | 0.0% | 45 | 0.0% | 0 | 0.0% | 8 | 0.0% | 0 | 0.0% | 2 | 0.0% | 0 | 0.0% | 10 | 0.0% |
|  | Regimen | Anthracycline | 209 | 7.9% | 31098 | 9.0% | 49 | 20.1% | 12563 | 27.6% | 7 | 17.5% | 7056 | 25.7% | 19 | 23.2% | 13289 | 28.8% |
|  |  | Taxane | 146 | 5.5% | 23532 | 6.8% | 47 | 19.3% | 11658 | 25.6% | 4 | 10.0% | 6927 | 25.2% | 14 | 17.1% | 10597 | 23.0% |
|  |  | Trastuzumab | 12 | 0.5% | 3088 | 0.9% | 117 | 48.0% | 25859 | 56.9% | 16 | 40.0% | 15935 | 58.1% | 1 | 1.2% | 707 | 1.5% |
| Radiation |  | Yes | 367 | 13.9% | 161755 | 46.8% | 44 | 18.0% | 17517 | 38.5% | 5 | 12.5% | 8584 | 31.3% | 14 | 17.1% | 17818 | 38.6% |
|  |  | No | 2209 | 83.8% | 173984 | 50.4% | 193 | 79.1% | 26404 | 58.1% | 31 | 77.5% | 17960 | 65.4% | 66 | 80.5% | 26808 | 58.1% |
|  |  | Missing | 61 | 2.3% | 9555 | 2.8% | 7 | 2.9% | 1545 | 3.4% | 4 | 10.0% | 898 | 3.3% | 2 | 2.4% | 1518 | 3.3% |
| Total (neoadjuvant + adjuvant) | Endocrine therapy | Yes | 2209 | 83.8% | 293118 | 84.9% | 177 | 72.5% | 30898 | 68.0% | 3 | 7.5% | 1267 | 4.6% | 3 | 3.7% | 2450 | 5.3% |
|  |  | No | 223 | 8.5% | 48266 | 14.0% | 64 | 26.2% | 16335 | 35.9% | 22 | 55.0% | 24040 | 87.6% | 49 | 59.8% | 39576 | 85.8% |
|  |  | Missing | 0 | 0.0% | 44 | 0.0% | 0 | 0.0% | 15 | 0.0% | 0 | 0.0% | 4 | 0.0% | 0 | 0.0% | 15 | 0.0% |
|  | Chemotherapy | Yes | 506 | 19.2% | 81963 | 23.7% | 115 | 47.1% | 27463 | 60.4% | 16 | 40.0% | 17179 | 62.6% | 47 | 57.3% | 32268 | 69.9% |
|  |  | No | 1926 | 73.0% | 259418 | 75.1% | 126 | 51.6% | 19876 | 43.7% | 9 | 22.5% | 8130 | 29.6% | 5 | 6.1% | 9763 | 21.2% |
|  |  | Missing | 0 | 0.0% | 47 | 0.0% | 0 | 0.0% | 9 | 0.0% | 0 | 0.0% | 2 | 0.0% | 0 | 0.0% | 10 | 0.0% |
|  | Chemotherapy regimen | Anthracycline | 288 | 10.9% | 47711 | 13.8% | 66 | 27.0% | 17296 | 38.0% | 12 | 30.0% | 10539 | 38.4% | 26 | 31.7% | 21038 | 45.6% |
|  |  | Taxane | 217 | 8.2% | 39077 | 11.3% | 63 | 25.8% | 16922 | 37.2% | 8 | 20.0% | 10930 | 39.8% | 21 | 25.6% | 17796 | 38.6% |
|  |  | anti-HER2 | 17 | 0.6% | 5159 | 1.5% | 130 | 53.3% | 30664 | 67.4% | 19 | 47.5% | 19905 | 72.5% | 2 | 2.4% | 1306 | 2.8% |
|  |  | Other | 17 | 0.6% | 2823 | 0.8% | 1 | 0.4% | 820 | 1.8% | 0 | 0.0% | 666 | 2.4% | 2 | 2.4% | 1522 | 3.3% |
